# Prevention of siderophore- mediated gut-derived sepsis due to *P. aeruginosa *can be achieved without iron provision by maintaining local phosphate abundance: role of pH

**DOI:** 10.1186/1471-2180-11-212

**Published:** 2011-09-26

**Authors:** Kathleen Romanowski, Alexander Zaborin, Hoylan Fernandez, Valeriy Poroyko, Vesta Valuckaite, Svetlana Gerdes, Donald C Liu, Olga Y Zaborina, John C Alverdy

**Affiliations:** 1 Department of Surgery, the University of Chicago, 5841 S. Maryland, Chicago, IL 60637, USA; 2 Fellowship for Interpretation of Genomes, 15w155 81st, Burr Ridge, IL 60527, USA; 3 Department of Surgery, St. Joseph's Hospital and Medical Center, 350 West Thomas Rd, Phoenix, AZ 85013, USA

## Abstract

**Background:**

During extreme physiological stress, the intestinal tract can be transformed into a harsh environment characterized by regio- spatial alterations in oxygen, pH, and phosphate concentration. When the human intestine is exposed to extreme medical interventions, the normal flora becomes replaced by pathogenic species whose virulence can be triggered by various physico-chemical cues leading to lethal sepsis. We previously demonstrated that phosphate depletion develops in the mouse intestine following surgical injury and triggers intestinal *P. aeruginosa *to express a lethal phenotype that can be prevented by oral phosphate ([Pi]) supplementation.

**Results:**

In this study we examined the role of pH in the protective effect of [Pi] supplementation as it has been shown to be increased in the distal gut following surgical injury. Surgically injured mice drinking 25 mM [Pi] at pH 7.5 and intestinally inoculated with *P. aeruginosa *had increased mortality compared to mice drinking 25 mM [Pi] at pH 6.0 (p < 0.05). This finding was confirmed in *C. elegans*. Transcriptional analysis of *P. aeruginosa *demonstrated enhanced expression of various genes involved in media alkalization at pH 6.0 and a global increase in the expression of all iron-related genes at pH 7.5. Maintaining the pH at 6.0 via phosphate supplementation led to significant attenuation of iron-related genes as demonstrated by microarray and confirmed by QRT-PCR analyses.

**Conclusion:**

Taken together, these data demonstrate that increase in pH in distal intestine of physiologically stressed host colonized by *P. aeruginosa *can lead to the expression of siderophore-related virulence in bacteria that can be prevented without providing iron by maintaining local phosphate abundance at pH 6.0. This finding is particularly important as provision of exogenous iron has been shown to have untoward effects when administered to critically ill and septic patients. Given that phosphate, pH, and iron are near universal cues that dictate the virulence status of a broad range of microorganisms relevant to serious gut origin infection and sepsis in critically ill patients, the maintenance of phosphate and pH at appropriate physiologic levels to prevent virulence activation in a site specific manner can be considered as a novel anti-infective therapy in at risk patients.

## Background

*Pseudomonas aeruginosa *is an opportunistic pathogen that is prevalent in the gut of hospitalized patients exposed to antibiotics and extreme physiologic stress such as major organ transplantation, injury, and sudden and severe insults [1-3]. *P. aeruginosa *is one of the most common causes of severe sepsis and its primary site of colonization and source of subsequent infection is the intestinal tract reservoir [3-5]. In previous work from our laboratory we analyzed multi-drug resistant isolates of *Pseudomonas aeruginosa *obtained from critically ill patients for their ability to disrupt the intestinal epithelial barrier and cause lethal gut-derived sepsis [6]. In these studies we identified that certain highly virulent and lethal isolates of *P. aeruginosa *respond to phosphate limitation by expressing outer surface appendages containing the phosphate signaling protein PstS [7]. We hypothesized that such responsiveness of these strains to phosphate limitation might have evolved from exposure to the depleted phosphate conditions present in a physiologically stressed host. We previously measured phosphate concentration in the intestine of mice following surgical injury and discovered that phosphate becomes rapidly depleted in the distal intestinal tract mucosa (cecum) and is associated with enhanced PstS expression in *P. aeruginosa *colonizing the mouse gut [8]. Further work using the prototype strain PAO1 demonstrated in both *C. elegans *and mice, that phosphate limitation causes activation of a lethal phenotype in *P. aeruginosa *that can be attenuated when local phosphate abundance/sufficiency is created via oral supplementation [9,10]. Molecular analysis of this response demonstrated that phosphate limitation activates a lethal phenotype in PAO1 via signaling mechanisms interconnecting phosphate acquisition systems (PstS-PhoB), quorum sensing (MvfR-PQS), and iron acquisition system (pyoverdin). We therefore hypothesized that maintenance of phosphate abundance/sufficiency at sites of *P. aeruginosa *colonization, such as the distal gut, may be a potential strategy to prevent virulence activation and hence mortality through the course of extreme physiologic stress when local phosphate stores become depleted.

Yet another important local microenvironmental cue that might affect the virulence and lethality of strains of *P. aeruginosa *that colonize the gut is pH. Measurements of luminal pH in the normal gastrointestinal tract have shown a progressive increase in pH from the duodenum to the terminal ileum, a decrease in the cecum, and then a slow rise along the colon to the rectum [11]. The relatively acidic pH range of 5.8-6.7 in the human proximal colon (cecum, right colon), the principle site of microbial colonization, has been repeatedly reported using various methods of pH analysis [12-15]. Importantly, pH has been found to be markedly increased in the proximal colon after severe insults such as sepsis, trauma, shock, and inflammatory bowel disease in human [1,11] as well as in mouse models of physiological stress induced by major surgery [16]. Yet whether changes in luminal pH correspond to changes within the colon mucosa, the primary site of a colonization and invasion of *P. aeruginosa *is unknown. As changes in pH in the proximal colon mucosa have the potential to affect the valence state and hence availability of both phosphate and iron to *P. aeruginosa *during intestinal colonization, the aims of the present study were to examine if pH changes in the proximal colon mucosa develop in mice following surgical injury that affect the ability of oral phosphate supplementation to protect against lethal sepsis due to intestinal *P. aeruginosa*.

## Methods

### Bacterial strains

Studies were performed with *P. aeruginosa *PAO1 strains obtained from two laboratories, MPAO1 (B. Iglewski, the original strain used to create the transposon mutant library at the University of Washington), and CorPAO1 (P. Cornelis), as well as with the CorPAO1 derivative mutant ΔPvdD/ΔPchEF.

### Mouse model of lethal gut-derived sepsis

Animal experiments were approved by the Animal Care and Use Committee at the University of Chicago (IACUC protocol 71744). Male C57BL6/HSD mice weighing 18 to 22 g were used for all experiments. Gut-derived sepsis was modeled by performing a 30% surgical left lateral hepatectomy with simultaneous injection of 10^7 ^CFU *P. aeruginosa *into cecum of mice pre-fasted 18 hours prior to surgery as previously described [16]. Mice were allowed access to either tap water, or 25 mM potassium phosphate-buffer (PB) pH 7.5, or 25 mM PB pH 6.0 through over the course of the experimental period.

### Measurement of intestinal mucosal pH

Intestinal mucosa (overlying mucus and intestinal epithelial cells) pH was measured with phenol red. Following 24 hrs after surgery, mice were sacrificed, and distal intestine of mice was harvested from rectum to jejunum, gently washed with water to remove loose luminal contents and then stained by flashing 5 times with 0.4% phenol red in buffer (0.145 M NaCl, 0.002 M KH_2_PO_4_, 0.003 M Na_2_HPO_4_). The intestine was opened longitudinally and mucosal pH measured semi-quantitatively using pH standards stained with phenol red.

### *C. elegans *model

*C. elegans *killing assays were performed as we previously reported [9] with modifications. Briefly, *P. aeruginosa *PAO1 grown on solid TSB was collected and suspended in either 25 mM potassium phosphate buffer (PB), pH 6.0 or PB pH 7.5 to a 30 μl volume that was poured on NGM agarized media (peptone, 2.5 g/L; NaCl, 3 g/L; MgSO_4_, 1 mM; CaCl_2_, 1 mM; agar 17 g/L) supplemented with 25 mM PB pH 6.0 or pH 7.5, respectively. PAO1 lawns were grown during 24 hrs at 37°C following overnight incubation at room temperature, and then were used for feeding *C. elegans*. As a control of phosphate limitation, *P. aeruginosa *PAO1 lawns were prepared on NGM containing 0.1 mM PB, pH6.0. Pre-fasted worms were transferred onto lawns and mortality followed for up to 60 hrs.

### Genome-wide transcriptional analysis

All samples for gene expression analysis were prepared in triplicate. *P. aeruginosa *MPAO1 cells collected from lawns grown on NGM/[Pi]25 mM, pH 6.0 or NGM/[Pi]25, pH 7.5 were used for RNA isolation as previously described. Microarray analysis was performed using Affymetrix *P. aeruginosa *GeneChips (Affymetrix, Santa Clara, CA) at the University of Chicago Functional Genomics Facility and data were analyzed as previously described [9]. Microarray data were deposited in GEO database, accession number GSE29789.

### QRT-PCR analysis

Multiplex qRT-PCR was performed to simultaneously analyze the expression of selected genes in *P. aeruginosa *MPAO1 grown under pH 6.0 and pH 7.5 in NGM-Pi 25 mM. Gene clusters for the analysis were chosen as representatives of phosphate signaling and acquisition, quorum sensing, and iron acquisition. Overnight *P. aeruginosa *MPAO1 culture was diluted 1:50 in triplicate in 25 mM phosphate NGM media at pH 6.0 and 7.5, and grown for 9 hrs at 37°C. RNA was isolated and reversed to cDNA as previously described [7]. QRT-PCR analysis was performed as previously described [9]. Briefly, gene specific primers (Tm = 60°C) to amplify 100 bp fragments of target mRNA were designed based on *in silica *analysis for amplification specificity by BLAST search against the database of *P. aeruginosa *PAO1 genome. Gene expression was normalized to *tpiA *(PA4748) whose expression was not influenced by pH in microarray analysis, and which was used in our previous QRT-PCR analyses [9]. Fold changes of expression levels were determined by normalization to expression at pH 6.0.

### Pyoverdin assay

Pyoverdin production was measured by fluorescence at 400 ± 10/460 ± 10 excitation/emission, and measurements of relative fluorescence units (RFU) were normalized to cell density units as absorbance at 600 nm in bacterial cultures growing in black, clear bottom 96-well plates (Corning Incorporated, Corning, NY, Costar 3603) using a 96-well Microplate Fluorimeter Plate Reader (Synergy HT, Biotek Inc., Winooski, VT). In the experiments with iron supplementation, pyoverdin was measured in supernatants by absorbance at 405 nm as previously described [17], and normalized to initial cell density.

### Iron concentration assay

Iron was measured using a Roche/Hitachi MOD P automated clinical chemistry analyzer using the FerroZine method (the minimum detection limit is 0.1 μg/ml). Results were reproduced in 3 biological replicates.

### Bioinformatics

Microarray data were analyzed using gene annotations provided by the SEED database http://www.theSEED.org/ and Pseudomonas Genome Database http://www.pseudomonas.com/.

### Statistical analysis

Statistical analysis of the data was performed with Student t-test using Sigma plot software, and Kaplan-Maier survival graphs using SPSS 18 software.

## Results

### Surgical injury (30% hepatectomy) increases the distal intestinal mucosal pH that can be maintained by pH adjusted oral phosphate supplementation

**Figure 1:**
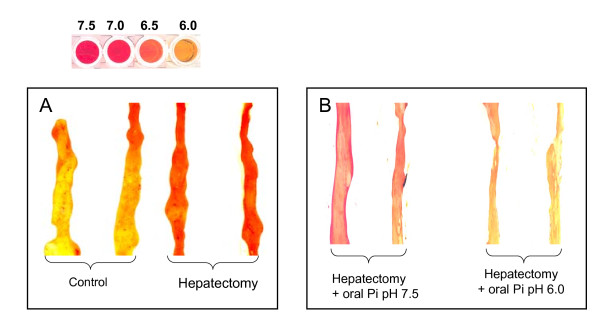
**Intestinal mucus pH**. Red phenol staining of (A) proximal colon of control and surgically stressed mice (30% hepatectomy), and (B) proximal colon of surgically stressed mice drinking 25 mM phosphate solution at pH 7.5 or pH 6.0. Experiments were performed in triplicate and representative images of the colon isolated and stained with 0.04% phenol red from 2 mice of each group are shown.

In order to determine whether the pH of the intestinal mucosa, the major colonization site of microbial pathogens, is affected by surgical injury, mucosal pH was measured using phenol red staining of intestinal segments of control and surgically injured mice. The pH of proximal colon segments, the densest region of microbial adherence, was measured in mice 22 hours following sham laparotomy or 30% hepatectomy. Results demonstrated pH shift from ~6.0 in sham mice to ~ 7.0-7.5 in mice subjected to 30% hepatectomy (Figure 1A). In mice drinking an oral ad libitum solution of 25 mM phosphate buffer adjusted to pH 6.0 or 7.5, intestinal mucosal pH in the proximal colon stabilized to the corresponding pH suggesting that, in mice, distal intestinal pH can be manipulated by oral pH adjustment (Figure 1B).

### Oral phosphate protects against the lethal effect of intestinal *P. aeruginosa *following surgical injury in a pH dependent manner

**Figure 2:**
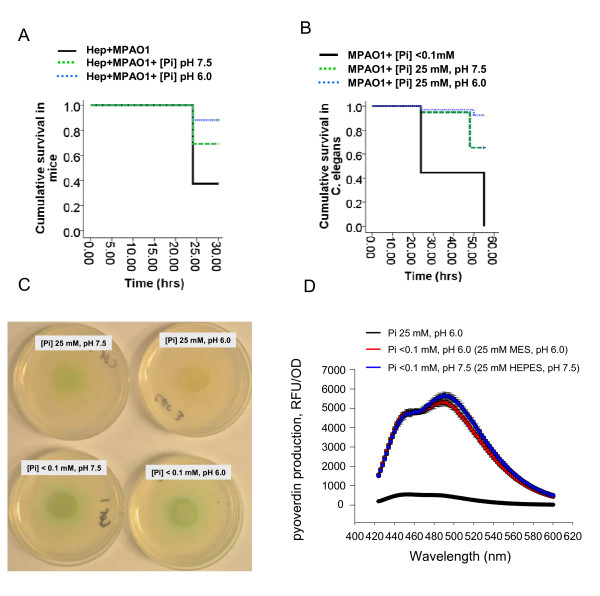
**Effect of pH on *P. aeruginosa *PAO1 virulence and pyoverdin production**. (A) Survival in mice subjected to hepatectomy and intestinal injection of *P. aeruginosa*. All mice were drank either water (var. Hep+MPAO1), 25 mM potassium phosphate buffer at pH 6.0 (var. Hep+MPAO1+[Pi] pH 6.0), or 25 mM potassium phosphate buffer at pH 7.5 (var. Hep+MPAO1+[Pi] pH 7.5). Results were reproduced in 3 experiments, n = 16/group, p < 0.05 in between pH7.5 and pH6.0 groups. (B) Survival in *C. elegans *feeding on *P. aeruginosa *PAO1 lawns. Results were reproduced in triplicate, n = 63/group, p < 0.05 in between pH7.5 and pH6.0 groups. (C) Pigmentation of *P. aeruginosa *PAO1 lawns grown at different phosphate and pH levels. The pH shift from 6.0 to 7.5 changes pigmentation on lawns containing Pi 25 mM. However, highly intense pigmentation is observed in *P. aeruginosa *PAO1 when grown as lawns at low (<0.1 mM) phosphate independent of pH. (D) The enhanced production of pyoverdin under conditions of phosphate limitation is not affected by pH changes.

We next determined the effect of pH on the expression of a lethal phenotype in intestinal *P. aeruginosa *using a model developed by our laboratory [16,18]. In this model, mice are subjected to an otherwise fully recoverable surgical injury (30% hepatectomy) with simultaneous injection of *P. aeruginosa *into the cecum which consistently results in > 60% mortality in 48 hr. In the present study, to generate negative controls, groups of mice were subjected to hepatectomy without injection of *P. aeruginosa *and drank either water, or 25 mM [Pi], pH 6.0, or 25 mM [Pi], pH 7.5 ad libitum (n = 16/group). No mice in any of these groups developed signs of sepsis or mortality at 48 hours and appeared completely healthy. In contrast, and consistent with our previous studies in this model [7-9], mice drinking water ad libitum and intestinally inoculated with *P. aeruginosa *PAO1 following surgical hepatectomy developed gross signs of sepsis (chromodacctyrrhea, ruffled fur, lethary, scant diarrhea) and a ~60% mortality rate at 48 hours. Mortality in mice intestinally inoculated with *P. aeruginosa *PAO1 following 30% hepatectomy and drinking 25 mM [Pi], pH 7.5 ad libitum was significantly attenuated (from 60% to 30%) with an even further mortality attenuation down to ~ 10% when mice drank 25 mM [Pi], pH 6.0 (Figure 2A).

In order to define the effect of pH on the lethality of *P. aeruginosa*, we used a more ordered host model system of *C. elegans *where worms feed on *P. aeruginosa *lawns grown at varying levels of phosphate and pH. Briefly, nematodes fed on *P. aeruginosa *lawns grown on agarized Nematode Growth Media (NGM) in which 25 mM potassium-phosphate buffer was adjusted to pH 6.0 or pH 7.5. Suspension of *P. aeruginosa *PAO1 to create the bacterial lawns was also prepared in 25 mM [Pi] at pH6.0 or 7.5 respectively to maintain consistency throughout the experimental period. As positive controls, parallel experiments were performed where worms fed on lawns of *P. aeruginosa *grown on low phosphate medium (0.1 mM) similar to our previously published experiments [9]. Results demonstrated that the killing effect of *P. aeruginosa *against *C. elegans *at high phosphate concentration was enhanced at pH 7.5 compared to 6.0 (Figure 2B). Importantly, low phosphate conditions induced the highest lethality rate consistent with our previous findings and demonstrated that extracellular phosphate is a major cue that activates virulence [9]. Previous work from our laboratory demonstrated that red material accumulated in the digestive tube of dying of *C. elegans *worms feeding on *P. aeruginosa *at low phosphate that consisted of the *P. aeruginosa *virulence-related quinolone signal PQS complexed with iron (PQS-Fe ^3+^). This complex was determined to be toxic to *C. elegans *especially when combined with rhamnolipids [9]. In the current study, the red material was not observed when *C. elegans *fed on *P. aeruginosa *PAO1 lawns grown at [Pi] 25 mM, pH 7.5 suggesting a lack of either PQS or pyoverdin production. The observation of yellow-green coloration suggested however that pyoverdin was produced at [Pi] 25 mM pH 7.5. It is important to note that the adjustment of pH did not affect the intense green coloration under low phosphate conditions suggesting that phosphate limitation is still a major factor for green pigment production (Figure 2C). Furthermore, enhanced pyoverdin production under conditions of phosphate limitation was not affected if pH is stabilized using 25 mM HEPES, pH7.5 or 25 mM MOPS, pH 6.0 (Figure 2D).

### A pH of 7.5 at high phosphate concentration (25 mM) induces the expression of iron starvation (IS) and ferrous uptake regulated (FUR) genes but not MvfR-PQS and results in expression of siderophore-mediated virulence in *P. aeruginosa*

We next performed a genome wide transcriptome analysis of PAO1 grown as lawns on NGM at pH 7.5 versus pH 6.0 (deposited in GEO database, accession number GSE29789) to more completely understand the virulence profile associated with *P. aeruginosa *lethality in the *C. elegans *model. Results demonstrated that a pH shift from 6.0 to 7.5 under conditions of phosphate abundance (25 mM) led to increased expression of all iron-dependent genes in *P. aeruginosa *PAO1 (Table 1). A significant (1.5-10.9 fold) increase in the expression of FUR regulated genes was observed suggesting that *P. aeruginosa *experiences intracellular iron insufficiency, perhaps owing to a relative decrease in iron solubility at a more alkaline pH. Among FUR regulated genes of interest was *pvdS *(PA2426) which encodes the sigma factor PvdS, a transcriptional regulator that controls the expression of the IS regulon including genes involved in the non-ribosomal biosynthesis of the siderophore pyoverdin, and the lethal toxin exotoxin A (*toxA*). Data demonstrated that *pvdS *itself as well as components of the PvdS-regulated iron siderophore sensor and receptor systems PA1911-1912, PA4895-4896, PA2467-2468, PA0471-0472, and *toxA *were overexpressed at pH7.5 compared to pH6.0. We initially assumed that the PstS-PhoB signaling/acquisition, which is normally activated under low phosphate conditions, might be paradoxically activated under high phosphate conditions at pH 7.5 if *P. aeruginosa *experienced relative phosphate limitation as a result of shift to a less soluble dibasic form. Lack of increased expression of PstS-PhoB in the analysis suggested however that both H_2_PO_4_^- ^and HPO_4_^2- ^are able to bind PstS and suppress the PHO regulon. The expression of quorum sensing genes including MvfR-PQS QS system was not increased at pH7.5 consistent with our previously published data demonstrating a regulatory role of phosphate on the MvfR-PQS signaling pathway beyond quorum sensing [9].

**Table 1:** *P. aeruginosa *genes with enhanced expression at pH 7.5 vs pH 6.0

PA ID	Gene name	Fold expression pH7.5 vs pH6.0	Regulon	Function	Subsystem
PA1134		2.58	IS	probable membrane protein	

PA1148	toxA	2.33	IS	exotoxin A precursor	

PA2384		4.78		Hypothetical protein in pyoverdin gene cluster/Fe2+/Zn2+ uptake regulation proteins	Siderophore_Pyoverdine

PA2385	pvdQ	2.56	FUR	Acyl-homoserine lactone acylase PvdQ (EC 3.5.1.-), quorum-quenching	Siderophore_Pyoverdine

PA2386	pvdA	2.99	IS	L-ornithine 5-monooxygenase (EC 1.13.12.-), PvdA of pyoverdin biosynthesis	Siderophore_Pyoverdine

PA2389	pvdR	2.36	IS	pyoverdine-specific efflux macA-like protein	Siderophore_Pyoverdine

PA2390	pvdT	2.01	IS	Pyoverdine efflux carrier and ATP binding protein	Siderophore_Pyoverdine

PA2391	opmQ	1.86	IS	Outer membrane pyoverdine eflux protein	Siderophore_Pyoverdine

PA2392	pvdP	2.98	IS	Pyoverdine biosynthesis related protein PvdP, Twin-arginine translocation pathway signal domain	Siderophore_Pyoverdine

PA2393	pvdM	3.43	IS	Putative dipeptidase, pyoverdin biosynthesis PvdM	Siderophore_Pyoverdine

PA2394	pvdN	3.24	IS	Pyoverdin biosynthesis protein PvdN, putative aminotransferase, class V	Siderophore_Pyoverdine

PA2395	pvdO	2.00	IS	PvdO, pyoverdine responsive serine/threonine kinase	Siderophore_Pyoverdine

PA2396	pvdF	2.53	IS	Pyoverdine synthetase PvdF, N5-hydroxyornithine formyltransferase	Siderophore_Pyoverdine

PA2397	pvdE	3.16	IS	PvdE, pyoverdine ABC export system, fused ATPase and permease components	Siderophore_Pyoverdine

PA2398	fpvA	4.07	IS	Outer membrane ferripyoverdine receptor FpvA, TonB-dependent	Siderophore_Pyoverdine

PA2399	pvdD	3.62	IS	Pyoverdine sidechain non-ribosomal peptide synthetase PvdD	Siderophore_Pyoverdine

PA2400	pvdJ	3.84	IS	Pyoverdine sidechain non-ribosomal peptide synthetase PvdJ	Siderophore_Pyoverdine

PA2402	pvdI	4.22	IS	Pyoverdine sidechain non-ribosomal peptide synthetase PvdI	Siderophore_Pyoverdine

PA2403		4.62		Putative iron-regulated membrane protein	Siderophore_Pyoverdine

PA2404		4.96		Putative thiamine pyrophosphate-requiring enzyme	Siderophore_Pyoverdine

PA2405		5.71		Hypothetical protein in pyoverdin gene cluster	Siderophore_Pyoverdine

PA2406		3.84		Hypothetical protein in pyoverdin gene cluster	Siderophore_Pyoverdine

PA2407		2.34		Cation ABC transporter, periplasmic cation-binding protein, PA2407 homolog	Siderophore_Pyoverdine

PA2408		2.82		ABC transporter in pyoverdin gene cluster, ATP-binding component	Siderophore_Pyoverdine

PA2409		1.69		ABC transporter in pyoverdin gene cluster, permease component	Siderophore_Pyoverdine

PA2410		1.84		ABC transporter in pyoverdin gene cluster, periplasmic component	Siderophore_Pyoverdine

PA2411		2.98	IS	Probable thioesterase involved in non-ribosomal peptide biosynthesis, PA2411 homolog	Siderophore_Pyoverdine

PA2412		3.12	IS	Hypothetical MbtH-like protein	Siderophore_Pyoverdine

PA2413	pvdH	3.04	IS	Pyoverdin biosynthesis protein PvdH, L-2, 4-diaminobutyrate:2-oxoglutarate aminotransferase	Siderophore_Pyoverdine

PA2424	pvdL	3.20	IS	Pyoverdine chromophore precursor synthetase PvdL	Siderophore_Pyoverdine

PA2425	pvdG	4.07	IS	Thioesterase PvdG involved in non-ribosomal peptide biosynthesis	Siderophore_Pyoverdine

PA2426	pvdS	5.21	FUR	Sigma factor PvdS, controling pyoverdin biosynthesis	Siderophore_Pyoverdine

PA2427		6.13	IS	Hypothetical protein PvdY	Siderophore_Pyoverdine

PA4168	fpvB	2.03		Outer membrane ferripyoverdine receptor FpvB, for Type I pyoverdine	Siderophore_Pyoverdine

PA5150		2.44	IS	probable short-chain dehydrogenase	

PA0471	fluR	2.75	FUR	probable transmembrane sensor	

PA0472	fluI	2.59	FUR	probable sigma-70 factor, ECF subfamily	

PA0672	hemO	3.61	FUR	Heme oxygenase HemO, associated with heme uptake	Hemin_transport_system

PA2467	foxR	2.08	FUR	Fe2+-dicitrate sensor, membrane component	

PA2468	foxI	2.86	FUR	probable sigma-70 factor, ECF subfamily	

PA4227	pchR	4.73	FUR	Transcriptional regulator PchR	Siderophore_pyochelin

PA4467		7.46	FUR	Metal transporter, ZIP family	

PA4468	sodM	5.59	FUR	Manganese superoxide dismutase (EC 1.15.1.1)	

PA4469		10.90	FUR	FOG: TPR repeat	

PA4470	fumC1	7.91	FUR	Fumarate hydratase class II (EC 4.2.1.2)	TCA_Cycle

PA4471		7.01	FUR	FagA protein	

PA4515		2.80	FUR	Iron-uptake factor PiuC	Transport_of_Iron

PA4516		1.87	FUR	FOG: TPR repeat, SEL1 subfamily	

PA4708	phuT	2.00	FUR	Heme-transport protein, PhuT	Hemin_transport_system

PA4709		2.22	FUR	probable hemin degrading factor	Hemin_transport_system

PA4710	phuR	2.00	FUR	Haem/Haemoglobin uptake outer membrane receptor PhuR precursor	Ton_and_Tol_transport_systems

PA4895		1.47	FUR	Iron siderophore sensor protein	Iron_siderophore_sensor_&_receptor_system

PA4896		3.14	FUR	probable sigma-70 factor, ECF subfamily	Iron_siderophore_sensor_&_receptor_system

PA1911	femR	3.55		sigma factor regulator, FemR	

PA1912	femI	5.53		ECF sigma factor, FemI	

**Figure 3:**
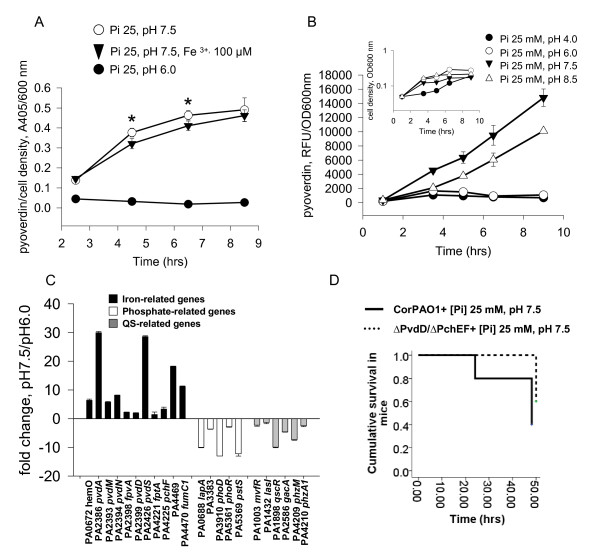
**Pyoverdin production is significantly increased at basic pH and plays a major role in the virulence of *P. aeruginosa***. (A) Production of pyoverdin normalized to cell density in *P. aeruginosa *PAO1 grown in liquid NGM at varying pH. n = 3, *p < 0.05 between Pi25 mM, pH 7.5 and Pi25 mM, pH7.5 +Fe^3+^, 100 μM. (B) Effect of pH changes on pyoverdin production and growth (inserted panel) in *P. aeruginosa *PAO1 at high Pi concentration (25 mM). (C) QRT-PCR demonstrating enhanced expression of iron-related but not phosphate- and QS-related genes. (D) PAO1 mutant deficient in the production of pyoverdin and pyochelin (ΔPvdD/ΔPchEF) is significantly attenuated in lethality in mice at pH 7.5. Mice were subjected to hepatectomy and intestinal injection with either wtPAO1 or its derivative mutant ΔPvdD/ΔPchEF. All mice were given 25 mM potassium phosphate buffered to pH 7.5 in their drinking water. Results were performed in duplicate. Cumulative survival is represented as Kaplan-Meyer survival curves, n = 10/group, p < 0.05, Log-Rank (Mantel-Cox).

While pyoverdin production is considered to be a quorum sensing related exoproduct of *P. aeruginosa *[19], our microarray results suggest that pH dependent expression of pyoverdin-related genes is not related to quorum sensing. To verify this, we dynamically measured *P. aeruginosa *PAO1 pyoverdin production during growth in liquid NGM media containing 25 mM [Pi] at pH 7.5 versus pH6.0. Results demonstrated that pyoverdin production was developed at 3 hrs of growth (Figure 3A) at 25 mM Pi, pH 7.5, and was partially suppressed by the addition of 100 μM Fe^3+^. Most notably, suppression of pyoverdin production at [Pi] 25 mM, pH 6.0 was significantly higher compared to that provided by iron supplementation at [Pi] 25 mM pH 7.5. The concentration of iron in both liquid media NGM Pi25 mM, pH 6.0 and NGM Pi25 mM, pH 7.5 was measured and found to be very low (< 0.1 μg/ml (< 1.78 μM)). Given that the concentration of iron needed to partially attenuate pyoverdin production in NGM Pi25 mM, pH 7.5 is as high as 100 μM (Figure 3A), we are confident that the pH, not the extracellular iron concentration, was a major factor leading to the triggering of pyoverdin production under conditions of similar extracellular iron concentration. Since iron binding to pyoverdin quenches its fluorescence, the pyoverdin production in these experiments was measured in supernatants by absorbance at 405 nm as previously described [17], and measurements were normalized to initial cell density. Results demonstrated that the expression of pyoverdin can be prevented without providing iron by maintaining local phosphate abundance at pH 6.0.

The effect of pH on pyoverdin production measured by fluorescence as previously described [9] was verified in the range of 4.0 to 8.5 (Figure 3B). Results demonstrated that the pyoverdin production is similar between pH4.0 and 6.0 (low level of pyoverdin), and between pH7.5 and 8.5 (high level of pyoverdin). We noticed however that the growth of *P. aeruginosa *at pH 4.0 was greatly delayed up to 4 hrs (Figure 3B, inserted panel). At this point, the pH of bacterial culture changed on its own from 4.0 to 5.5 and further changed to pH ~ 6.0 at 9 hrs. Bacteria significantly increased their growth rate at 9 hours. Alternatively, bacteria grew very well at pH 8.5, produced pyoverdin, and there was no change from the initial pH. This finding supports our hypothesis that *P. aeruginosa *can regulate its environmental pH to facilitate its colonization.

Next, we measured the expression of QS- and iron- related genes by qRT-PCR in *P. aeruginosa *PAO1 grown for 9 hrs in liquid NGM media at pH 7.5 versus 6.0. Gene expression was normalized to *tpiA *(PA4748) expression and then fold change was determined using expression of PAO1 measured in NGM at pH 6.0 as 100%. Results demonstrated increased expression of iron related genes and decreased expression of both quorum sensing and low phosphate- related genes at pH 7.5 versus 6.0 (Figure 3C). These data may confirm that pH-mediated expression of iron- regulated genes is not dependent on quorum sensing. However, we found significant down-regulation (10 fold) of the *qscR *gene encoding LuxR-type "orphan" receptor QscR, a potent QS repressor [20]. As down- regulation of *qscR *may modulate LasRI activity at the same level of *lasR *expression [21-23], we cannot completely exclude the role of quorum sensing in pyoverdin regulation. QscR shares affinity for lactone QS molecules with LasR and can form inactive heterodimers with LasR and RhlR monomers to negatively regulate QS. Therefore attenuation of QscR production could lead to LasRI-mediated expression of pyoverdin-related genes. Results from our microarray analysis performed on high cell density cells demonstrate that *qscR *was down-regulated (-1.55) while *lasR *(1.6 fold) was upregulated (GEO database, accession number GSE29789). Such subtle changes in the expression of transcriptional regulators LasR and QscR may have profound downstream effects and therefore we cannot reject or confirm a regulatory role of QS in pyoverdin production at pH 7.5.

Finally to confirm the critical role of siderophores on *P. aeruginosa *lethality induced at pH7.5, we performed reiterative experiments using the double mutant ΔPvdDΔPchEF in mice. Intestinal inoculation with ΔPvdDΔPchEF resulted in attenuated lethality in mice exposed to surgical injury suggesting that iron acquisition factors (i.e pyoverdin and pyochelin) play an important role in *P. aeruginosa *mortality when mice are orally supplemented with phosphate (Pi 25 mM) at pH 7.5 (Figure 3D).

### *P. aeruginosa *tends to alkalize medium at pH 6.0

Among the 126 genes that were up- regulated at pH 6.0, many appear to be associated with various cellular processes leading to media alkalization (Table 2). As case in point, expression of all genes of the arginine deiminase (ADI) pathway was enhanced 2.2 - 4.3 fold at pH 6.0. The ADI pathway has been well established as a counteracting agent in acidic environments such as those encountered by various pathogens [24]. This pathway is unique in that it allows regeneration of ATP from ADP without generating reduced NAD(P) and without medium acidification due to the fact that most of its fermentation end-products are gaseous. Furthermore, ammonia production as a result of activation of this pathway directly alkalinizes the medium. The 2.1 - 3.5-fold increase in the expression of the spermidine export protein mdtJI homolog (PA1541 - PA1540) might also contribute to medium alkalization since production and excretion of polyamines has been shown in *E. coli *to contribute to an increase in the pH of the extracellular medium [25,26]. Multiple genes of the denitrification chain were upregulated at pH 6.0 as well, including those encoding the 4 core enzymatic complexes (nitrate reductase NAR, nitrite reductase NIR, nitric oxide reductase NOR, and nitrous oxide reductase N_2_OR), as well as supporting components, such as protoheme and heme d1 biosynthetic genes. This observation is in agreement with the computation based prediction that microbial assimilation of 1 mole nitrate or nitrite results in increase of alkalinity by 1 mole [27]. These results may be unexpected if one considers nitrate respiration and arginine fermentation to be strictly anaerobic processes. However, it has been well established that *P. aeruginosa *is capable of performing denitrification at relatively high dissolved oxygen levels [28-30]. The physiological role for aerobic denitrification has not yet been fully elucidated. From a purely energetic standpoint, the advantage of co-respiration using both oxygen and nitrate is not obvious, since energetically denitrification is less efficient than aerobic respiratory pathways. However, this apparent paradox has been addressed in different bacteria and additional physiological roles have been suggested for various denitrification enzymes [31]. Our own analysis of global gene expression in *P. aeruginosa *in this study points to role of aerobic denitrification as a response to media acidification assuming that aerobic denitrification might be essential for *P. aeruginosa *to maintain an optimum pH during infection of the gut. Similarly, the role of arginine deiminase system is far more complex than merely to support cellular survival under anaerobiosis. In fact, the major function of this system in a variety of lactic acid bacteria and Streptococcal species has been shown to protect organisms against acid damage [24,32]. For *P. aeruginosa *this role has not been previously demonstrated and therefore is novel. Finally we observed attenuated expression of multiple stress-related and resistance-related genes at pH 7.5. Taken together these findings suggest that pH 7.5 is more physiologic for *P. aeruginosa *and that *P. aeruginosa *may regulate its environmental pH to facilitate its colonization and/or invasion.

**Table 2:** *P. aeruginosa *genes with decreased expression at pH 7.5 vs pH 6.0

PA ID	Gene name	Fold expression pH7.5 vs pH6.0	Function	Subsystem
PA5170	arcD	-1.91	Arginine/ornithine antiporter ArcD	Arginine deiminase pathway

PA5171	arcA	-4.3	Arginine deiminase (EC 3.5.3.6)	Arginine deiminase pathway

PA5172	arcB	-2.82	Ornithine carbamoyltransferase (EC 2.1.3.3)	Arginine deiminase pathway

PA5173	arcC	-2.13	Carbamate kinase (EC 2.7.2.2)	Arginine deiminase pathway

PA0530		-2.49	Acetylornithine aminotransferase (EC 2.6.1.11)	Arginine_Biosynthesis_extended

PA3865		-2.74	Arginine/ornithine ABC transporter, periplasmic arginine/ornithine binding protein	Arginine deiminase pathway

PA1540		-2.14	Spermidine export protein mdtI	Small_Multidrug_Resistance

PA1541		-3.44	Spermidine export protein mdtJ	Small_Multidrug_Resistance

PA0509	nirN	-3.39	Nitrite reductase associated c-type cytochorome NirN	Dissimilatory_nitrite_reductase

PA0510		-4.39	Uroporphyrinogen-III methyltransferase (EC 2.1.1.107)	Dissimilatory_nitrite_reductase

PA0511	nirJ	-5.67	Heme d1 biosynthesis protein NirJ	Dissimilatory_nitrite_reductase

PA0512		-1.84	Heme d1 biosynthesis protein NirH	Dissimilatory_nitrite_reductase

PA0513		-1.76	Heme d1 biosynthesis protein NirG	Dissimilatory_nitrite_reductase

PA0514	nirL	-2.32	Heme d1 biosynthesis protein NirL	Dissimilatory_nitrite_reductase

PA0515		-7.33	Heme d1 biosynthesis protein NirD	Dissimilatory_nitrite_reductase

PA0516	nirF	-2.59	Heme d1 biosynthesis protein NirF	Dissimilatory_nitrite_reductase

PA0517	nirC	-7.03	Cytochrome c55X precursor NirC	Dissimilatory_nitrite_reductase

PA0518	nirM	-10.01	Cytochrome c551 NirM	Dissimilatory_nitrite_reductase

PA0519	nirS	-8.9	Cytochrome cd1 nitrite reductase (EC:1.7.2.1)	Denitrification

PA0520	nirQ	-2.02	Nitric oxide reductase activation protein NorQ	Denitrification

PA0521		-1.91	Nitric oxide reductase activation protein NorE	Denitrification

PA0523	norC	-8.51	Nitric-oxide reductase subunit C (EC 1.7.99.7)	Denitrification

PA0524	norB	-9.78	Nitric-oxide reductase subunit B (EC 1.7.99.7)	Denitrification

PA0525		-3.39	Nitric oxide reductase activation protein NorD	Denitrification

PA1172	napC	-1.51	Cytochrome c-type protein NapC	Nitrate_and_nitrite_ammonification

PA1173	napB	-2.01	Nitrate reductase cytochrome c550-type subunit	Nitrate_and_nitrite_ammonification

PA1174	napA	-2.01	Periplasmic nitrate reductase precursor (EC 1.7.99.4)	Nitrate_and_nitrite_ammonification

PA2662		-1.90	NnrS protein involved in response to NO	Denitrification

PA3391	nosR	-2.17	Nitrous oxide reductase maturation protein NosR	Denitrification

PA3392	nosZ	-3.16	Nitrous-oxide reductase (EC 1.7.99.6)	Denitrification

PA3393	nosD	-1.40	Nitrous oxide reductase maturation protein NosD	Denitrification

PA2826		-5.48	Glutathione peroxidase family protein	Stress response

PA2850		-2.28	Organic hydroperoxide resistance protein	Stress response

PA3017		-1.56	Universal stress protein UspA and related nucleotide-binding proteins	Stress response

PA3309		-3.47	Universal stress protein UspA and related nucleotide-binding proteins	Stress response

PA4352		-7.28	Universal stress protein UspA and related nucleotide-binding proteins	Stress response

PA5027		-4.50	Universal stress protein UspA and related nucleotide-binding proteins	Stress response

PA4760	dnaJ	-2.02	Chaperone protein DnaJ	Stress response

PA4761	dnaK	-2.41	Chaperone protein DnaK	Stress response

PA4762	grpE	-2.70	Heat shock protein GrpE	Stress response

PA4587	ccpR	-12.82	Cytochrome c551 peroxidase (EC 1.11.1.5)	Stress response

PA4206		-3.50	Probable Co/Zn/Cd efflux system membrane fusion protein	Resistance

PA4207		-3.52	RND multidrug efflux transporter; Acriflavin resistance protein	Resistance

PA4208		-3.52	Probable outer membrane efflux protein precursor	Resistance

### Comparative analysis of iron-related subsystems during phosphate limitation and a pH shift from 6.0 to 7.5 reveals the significant protective effect of phosphate supplementation

**Figure 4:**
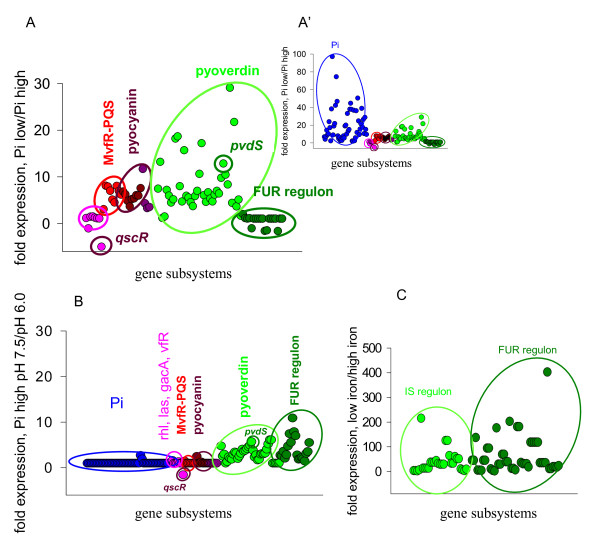
**The effect of phosphate and pH on the expression of pyoverdin-related genes.** (A, A') Transcriptional pattern response of *P. aeruginosa *PAO1 to phosphate limitation (< 0.1 mM) displayed at different scales: (A) in the absence of phosphate-related genes and (A') in the presence of phosphate-related genes. Pattern was drawn based on the results of Zaborin et al., 2009. (B) Transcriptional pattern response of *P. aeruginosa *PAO1 to a pH shift from 6.0 to 7.5 during phosphate sufficiency (25 mM). Pattern was drawn based on the current data. (C) Transcriptional response of IS (mainly pyoverdin-related genes) and FUR regulon in *P. aeruginosa *PAO1 during iron limitation. Pattern was drawn based on the results of Ochsner et al., 2002. Light green dots represent the fold expression in pyoverdin-related genes; dark green dots - FUR-regulated genes. The dark green circle surrounding *pvdS *indicates that this gene is regulated by FUR. The brown spots indicate genes involved in pyocyanin biosynthesis, red spots indicate genes belonging to MvfR and MvfR-regulated *pqsABCDE *operon, and pink spots indicate genes of quorum sensing regulatory elements such as *rhlI, rhlR, lasI, lasR, gacA, vfR, qscR*. The dark circle surrounding *qscR *indicates that this gene is involved in the regulation of pyocyanin biosynthesis. Blue spots in the panel A' represent phosphate-related genes.

We have previously shown that phosphate limitation induces three global virulence subsystems in *P. aeruginosa *PAO1 that include 1.) phosphate signaling/acquisition, 2.) MvfR-PQS of the core quorum sensing pathway and downstream regulated genes such as those involved in the biosynthesis of pyocyanin, and 3.) pyoverdin-related genes (Figure 4A, A') (Microarray data for phosphate limitation are deposited in GEO database, GEO accession number GSE30967). The upregulation of pyoverdin by phosphate limitation was surprising given that the expression of pyoverdin genes is regulated by the transcriptional regulator PvdS that by itself is part of the FUR regulon, and as such the expression of PvdS and its regulated genes strongly depends on iron concentration. One would assume that there is going to be more iron available at lower concentrations of phosphate since phosphate causes precipitation of iron, thereby decreasing its effective concentration. Indeed, the absence of activation of FUR-regulated genes (normally suppressed at high concentration of iron) suggested that iron was available for *P. aeruginosa *(Figure 4A) indicating that the response of *P. aeruginosa *at differing levels of Pi is not simply a matter of the interaction of iron and phosphate, but rather involves more complex yet- to- be elucidated mechanisms. Alternatively, the expression of pyoverdin genes and FUR regulon in high phosphate media at pH 7.5 (Figure 4B) demonstrated that *P. aeruginosa *was exposed to iron limiting conditions. Comparison of the signature of iron related genes during pH shift to 7.5 to that induced by iron limitation as reported by Ochsner et. al. [33] (Figure 4C) confirmed that *P. aeruginosa *experiences iron limitation at pH 7.5. Importantly, providing phosphate at pH 6.0 suppressed the expression of iron-related genes indicating a significant protective effect of phosphate supplementation at pH6.0.

## Discussion

Results from the present study build on our previous work to understand how local cues within the intestinal tract reservoir affect the virulence of colonizing pathogens that are capable of causing lethal gut- derived sepsis. Microbes that colonize the gut following extreme medical interventions such as major organ transplantation are under an unprecedented level of pressure to adapt to an highly abnormal environment in which pH is shifted, nutrient resources are limited, and the normal microbial flora is dramatically altered by the combined effects of extreme physiologic stress and antibiotic treatment. In this regard, the human opportunistic pathogen *P. aeruginosa *has been shown to rapidly colonize such patients and be a major primary source of infection and sepsis [34]. In many cases of severe sepsis the primary pathogen remains unidentified. In this regard, intestinal *P. aeruginosa *is particularly suited to use the intestinal tract as a privileged site with its unique ability to survive, persist, and mount a toxic offensive without extraintestinal dissemination (gut-derived sepsis) [35]. The emergence of pan-resistant strains of *P. aeruginosa *that often colonize the gut of the most critically ill patients begs the development of a non- antibiotic based approach that can suppress virulence activation of *P. aeruginosa *through the course of surgery or immuno-suppression as a containment rather than elimination strategy. To achieve this, a more complete understanding of the physico-chemical cues that characterize colonization sites of intestinal pathogens in critically ill patients is needed.

Our previous work suggests that a major environmental cue that shifts *P. aeruginosa *to express a lethal phenotype within the intestinal tract of surgically injured mice is the mucosal phosphate. During surgical injury, phosphate becomes depleted within the intestinal mucus and signals *P. aeruginosa *to express a lethal phenotype via pathways that triangulate three global virulence subsystems: phosphate signaling and acquisition, MvfR-PQS of quorum sensing, and pyoverdin production [9]. Importantly, maintenance of phosphate abundance/sufficiency via oral supplementation prevents activation of these pathways and attenuates mortality in mice and *C. elegans*. Results from the present study emphasize the importance of pH on the ability of phosphate to protect mice and *C. elegans *from the lethal effect of intestinal *P. aeruginosa*. This is particularly important given the observation that pH in the distal intestinal tract is increased in response to surgical injury. We focused on pH changes in the proximal colon (cecum) as it is the densest site of microbial colonization and the site of greatest immune activation in response to intestinal pathogens [36-40]. In addition, various reports confirm that experimental injury or human critical illness results in a similar shift in distal intestinal pH from a normal value of 6 to > 7 in both animals and humans [1,11,16]. Therefore the transcriptional response of *P. aeruginosa *PAO1 when the pH is shifted from 6.0 to 7.5 may have particular relevance *in vivo*.

Microarray and qRT-PCR analysis demonstrated the upregulation of all iron-regulated genes including pyoverdin-related ones at pH7.5 but did not demonstrate an increase in the expression of the quorum sensing system suggesting that iron acquisition is the main virulence feature of *P. aeruginosa *under these conditions. Interestingly, the expression pattern of other genes at pH 6.0 compared to 7.5 demonstrated the increased expression of multiple genes associated with cellular processes involved in media alkalization including expression of denitrification genes in *P. aeruginosa *which, to our knowledge, has not been previously reported. Finally we observed attenuated expression of multiple stress-related and resistance-related genes at pH 7.5. Taken together these findings suggest that pH7.5 is more physiologic for *P. aeruginosa *and that *P. aeruginosa *may regulate its environmental pH to facilitate its colonization and/or invasion being well equipped with multiple siderophores. Thus, these data provide one more example that demonstrates the connectedness of the metabolic and virulence response in *P. aeruginosa*. As a result of exposure to physiologic cues present in post-surgical patients, intestinal *P. aeruginosa *may be activated to alkalinize its local microenvironment which itself will lead to less iron availability and hence enhanced virulence. Thus a preventative strategy to maintain the intestinal pH at a more suitable level that suppresses virulence activation in problematic colonizing pathogens such as *P. aeruginosa *should be considered.

Data from the present study suggest that suppression of siderophore-related virulence expression in *P. aeruginosa *can be achieved without the need to provide iron by creating conditions of local phosphate sufficiency at pH6.0. This finding may be particularly important as provision of exogenous iron has been shown to have untoward effects when administered to critically ill and septic patients [41-43]. Iron administration has been shown to impair neutrophils function, increase the incidence of infections, and cause hemodynamic compromise in critically ill patients [41,44-47]. Data from the present study suggest that maintenance of phosphate and pH at appropriate physiologic levels prevents virulence activation in a site specific manner and as such, is an example of a non- antibiotic, anti-virulence based strategy to suppress the lethality of highly virulent pathogens such as *P. aeruginosa*. Given that phosphate, pH, and iron are near universal cues that suppress/activate the virulence of a broad range of microorganisms relevant to serious gut origin infection and sepsis in critically ill patients, a more complete understanding of how these elements can be controlled in a site specific manner through the course of extreme physiologic stress could led to novel anti-infective therapies in at risk patients.

## Conclusion

The GI tract expresses a highly variable pH that is region dependent and is affected by various physiologic conditions such as ischemia and the use of acid suppressing agents and other drugs employed during the treatment of critically ill patients. A shift in pH to ~7.5 in the intestinal mucus during physiological stress can lead to activation of multiple siderophore-related genes that directly impact microbial virulence. We show for the first time that suppression of siderophore-related virulence expression in *P. aeruginosa *can be achieved without providing iron by creating conditions of local phosphate sufficiency at pH 6.0. These findings may have significant therapeutic implications given that there is reluctance to provide excess iron in the face of life threatening infection. Understanding the local cues that activate virulence of common pathogens that colonize the gut during critical illness may lead to new insight into their pathogenesis.

## Authors' contributions

KR carried out measurements of intestinal mucosal pH, ran mice experiments, and measured iron concentration; AZ carried out *C. elegans *experiments, RNA isolation and preparation for microarray analysis, and performed pyoverdin assays; HF conceived of the study, measured intestinal mucus pH, and pyoverdin production; VP performed RT-PCR analysis; VV ran mice experiments; SG participated in the reconstruction of the microarray data to reveal main affected subsystems; DL conceived of the study, and participated in its design; OZ conceived of the study, participated in its design and coordination, performed microarray analysis, and wrote the manuscript; JA coordinated the study, participated in the design, and wrote the manuscript. All authors read and approved the final manuscript.
